# Immune responses to Tilapia lake virus infection: what we know and what we don’t know

**DOI:** 10.3389/fimmu.2023.1240094

**Published:** 2023-08-09

**Authors:** Japhette E. Kembou-Ringert, Dieter Steinhagen, Kim D. Thompson, Janet M. Daly, Mikolaj Adamek

**Affiliations:** ^1^ Department of Infection, Immunity and Inflammation, Great Ormond Street Institute of Child Health, University College London, London, United Kingdom; ^2^ Fish Disease Research Unit, Institute for Parasitology, University of Veterinary Medicine Hannover, Hannover, Germany; ^3^ Moredun Research Institute, Pentlands Science Park, Penicuik, United Kingdom; ^4^ School of Veterinary Medicine and Science, University of Nottingham, Sutton Bonington, United Kingdom

**Keywords:** Tilapia lake virus, immunity, innate immunity, adaptive immunity, antiviral response, host immune resistance, immune subversion

## Abstract

Tilapia lake virus (TiLV) is a novel contagious pathogen associated with a lethal disease affecting and decimating tilapia populations on several continents across the globe. Fish viral diseases, such as Tilapia lake virus disease (TiLVD), represent a serious threat to tilapia aquaculture. Therefore, a better understanding of the innate immune responses involved in establishing an antiviral state can help shed light on TiLV disease pathogenesis. Moreover, understanding the adaptive immune mechanisms involved in mounting protection against TiLV could greatly assist in the development of vaccination strategies aimed at controlling TiLVD. This review summarizes the current state of knowledge on the immune responses following TiLV infection. After describing the main pathological findings associated with TiLVD, both the innate and adaptive immune responses and mechanisms to TiLV infection are discussed, in both disease infection models and *in vitro* studies. In addition, our work, highlights research questions, knowledge gaps and research areas in the immunology of TiLV infection where further studies are needed to better understand how disease protection against TiLV is established.

## 1 Introduction

Tilapia lake virus (TiLV) or *Tilapia tilapinevirus* is an enveloped icosahedral virus of 55–75 nm (1), belonging to the *Amnoonviridae* family, and is characterized by a 10,323 kb segmented, negative sense, and single-stranded RNA (ssRNA) genome (2). TiLV is currently the sole representative member of this virus family (3), although recent meta-transcriptomic and data mining studies have identified several viral segments and transcripts related to TiLV PB1 gene (4, 5), that probably belong to novel divergently TiLV-related viruses. The TiLV genome is composed of ten ribonucleoproteins (RNP) units and encodes at least 14 predicted proteins (6), including the recently identified NP protein encoded by segment 4 (7).

TiLV primarily infects tilapia species [particularly Nile tilapia *Oreochromis niloticus*, Mozambique tilapia *O. mossambicus*, Gray tilapia (*Oreochromis niloticus x O. aureus*) and Red tilapia *Oreochromis* spp.)], although other fish species such as tinfoil barbs (*Barbonymus schwanenfeldii*) (8, 9), giant gourami *Osphronemus* (10), angelfish (*Pterophyllum scalare*) and firemouth cichlid (*Thorichthys meeki*) (11) have also shown susceptibility to TiLV infection and could be experimentally infected with the virus. TiLV clinical infection has also been experimentally recapitulated through intraperitoneal (IP) injection of adult zebrafish (*Danio rerio*) (12, 13), zebrafish larvae (14), juvenile rainbow trout (*Oncorhynchus mykiss*) and brown trout (*Salmo trutta*) (15).

Cases of co-infection of TiLV with *Aeromonas hydrophila* and *Streptococcus agalactiae* in farmed red hybrid tilapia have also been reported (16). In general, co-infections with TiLV and *Aeromonas* spp. seem to be frequent, and infection with TiLV appears to promote secondary bacterial infections, especially with *Aeromonas veronii* (17) and *Aeromonas hydrophila* (18). Together with sporadic *Streptococcus agalactiae* co-infections, these bacteria co-infections can synergistically increase fish mortality and worsen disease severity in affected tilapia (16–18). Indeed, TiLVD can cause mortalities as high as 90% in affected fish populations (19), even though a few cases of inapparent infection have also been documented (20).

The virus has a broad tissue tropism and can induce a systemic infection. Tissue tropism studies have shown the presence of the virus in multiple organs, including the brain, liver, kidney, muscles, gills, fins, spleen, intestines, eye, heart, ovaries and testis (21). Moreover, all the life stages of tilapia, including fertilized eggs, yolk-sac larvae, fries, fingerlings, and adults appear to be susceptible to TiLV (21–24) and vertical transmission from broodstock to progeny can also occur (25, 26), altogether making TiLV a significantly lethal pathogen.

The elimination of virus pathogens such as TiLV during infection largely depends on the presence of a functional immune system. In bony fish such as tilapia, the host innate immune system, which is at the forefront of fish immune defenses, activates and triggers antiviral and pro-inflammatory responses early during infection (27). The adaptive immune response, although often delayed, also plays a critical role in the clearance of viral pathogens during the later stages of infection and is essential for long-lasting immunity and a key factor in successful vaccination (28).

It has been shown that tilapia mount a protective immune response following exposure to TiLV (29), as around 200 differently expressed microRNAs regulating genes involved in the immune response have been identified in tilapia fish infected with TiLV (30). Moreover, over 4640 genes, some of which are involved in antigen processing and presentation, nuclear factor kappa-light-chain-enhancer of activated B-cells (NF-κB), interferon (IFN) and chemokine signaling, were found to be differentially expressed in the liver of tilapia experimentally infected with TiLV (31), all suggestive of an attempt to establish an antiviral state.

However, it has also been shown that TiLV can downplay the innate immune response, especially during the early stages of infection (32), suggesting the existence of yet to be discovered viral effector proteins involved in and associated with immune response modulation.

The development of effective therapeutics and prevention strategies against viral diseases certainly requires an understanding of the various immunopathogenesis processes and mechanisms occurring during viral infections and contributing to disease establishment and persistence. Although also associated with the damages caused by viral replication (viral related factors), disease pathogenesis following viral infections often appears to result from an abnormal host response or overreaction of the immune system (host-related factors) to resolve the infection. Given that several studies aimed at elucidating the immune responses occurring during TiLV infection have recently emerged, it is timely to review our current understanding of the mechanisms governing the antiviral response to TiLV infection as it is important for the development of novel drugs and antiviral treatment strategies for controlling TiLV infection.

## 2 Pathology of TiLV disease

The pathogenesis of TiLV is not yet fully defined and understood, partly because the cellular receptor for this virus has not yet been identified and its mode of entry is not yet fully resolved. From what is currently known, TiLV enters endothelial TmB cells via a dynamin-mediated endocytic pathway largely dependent on cholesterol rich lipid-rafts and cytoskeleton but not on clathrin (33). In addition, endosomal acidification seems to not be required for TiLV endosomal escape during virus entry (33).

### 2.1 TiLV tissue tropism

As previously mentioned, TiLV appears to exhibit a very broad tissue tropism as the virus is capable of replicating in the brain, liver, kidney, muscles, gills, fins, spleen, intestines, eye, heart, ovaries and testis (13, 34) of both infected tilapia and zebrafish, and immunohistochemical detection of TiLV using a TiLV immunoglobulin G antibody has revealed the presence of the virus in the endothelial cells of various organs (liver, pancreas, kidney, gills, intestines, brain, and spleen) as well as in the circulating leukocytes in the blood vessels (34).

Syncytia formation is a major pathological change reported by several studies during TiLVD (1, 17, 19, 24, 35–37). Although the mechanisms underlying this pathological finding have not yet been elucidated in the specific case of TiLV, several viruses are known to produce proteins capable of fusogenic activity (38, 39), and membrane fusion during infection with such viruses is a crucial step during virus entry of target cells. Although the fusion protein of TiLV remains to be identified and its fusogenic activity demonstrated, similar underlying fusion mechanisms as the ones described for some parainfluenza virus lineages (for which membrane fusion does not require low-pH) could be at play during TiLV infection. Moreover, the gene for RhoA, whose signaling has been associated with cell-to-cell fusion and syncytium formation during respiratory syncytial virus (RSV) infection (40), is upregulated in the liver during TiLV infection (31), suggesting that this small GTPase may play a role in initiating cell-to-cell fusion during TiLV infection. Further studies are thus required to shed some light on TiLV syncytia formation and the specific role of RhoA during this event.

### 2.2 Routes of infection and infection models

TiLV seems to have a relatively narrow host range with tilapia species being the canonical host for the virus. Therefore, the immune responses to TiLV infection have mainly been measured in Nile and red hybrid tilapia. Apart from the limited number of results obtained from infection performed by cohabitation, most results from experimental infections have been obtained following IP injection. This type of infection route does not allow the elucidation of the key antiviral responses at virus entry sites. Infection models based on IP injections of the canonical host often lead to a very fast onset of disease and mortality. In addition, TiLVD has also been modelled using an intragastric challenge model (35, 37). However, the low mortality rates observed with this route of infection (40% mortality after 10 days as opposed to 70% mortality in IP injected tilapia) may suggest a lack of systemic absorption from the digestive tract and raises the possibility that this route of infection is not the principal entry route of the virus in natural infection.

Other than tilapia, immune responses during TiLV infection have also been modelled in zebrafish which were also infected by IP injection (12, 13), or injection via duct of Cuvier of zebrafish larvae (14). In the zebrafish model, the virus has the ability to spread to several tissues of the body although it does not lead to high mortality (12, 13). Both zebrafish and tilapia are ray-finned fish and can tolerate tropical to sub-tropical water temperatures. Although most fish immune related genes are well-annotated in zebrafish (due to extensive studies conducted with zebrafish as an animal model) and can significantly inform our understanding of immune responses and pathways activated during TiLV infection, the zebrafish model remains limited by the requirement for IP-injection to initiate viral infection. Moreover, cases of natural infection of zebrafish with TiLV have not yet been reported suggesting the existence of factors restricting TiLV infection at virus entry sites in zebrafish, further emphasising that zebrafish is not a natural host for TiLV. As such, antigen recognition, disease establishment and progression, and immune responses and modulation might be different in this model.

## 3 Innate immune response

TiLV seems to be well recognized by pattern recognition receptors (PRRs) which elicit several key host immune responses such as increasing the release of antiviral factors important in restricting viral replication and spreading.

It is generally accepted that upon viral infection, the pathogen-associated molecular patterns (PAMPs) of viruses, either non-capped double or single stranded RNA (dsRNA or ssRNA) are sensed by cellular pattern recognition receptors (PRRs) located at the cell surface, in endosomes or in the cytosol. Sensing of viruses by PRRs such as Toll-like receptors (TLRs) and retinoic acid inducible gene I (RIG-I)-like receptors (RLRs) leads to the activation of several signaling pathways and transcription factors such as the interferon regulatory factors (IRFs) and NF-κB (41), both subsequently inducing the transcription of type I IFNs, crucial for the establishment of an antiviral state.

### 3.1 Activation of innate immune signaling upon intracellular detection of TiLV infection

It has been demonstrated that during TiLV infection, there is a significant upregulation of the PRR sensors TLR3 and TLR7 in the brain of TiLV-infected tilapia (31, 42). In contrast, upregulation of TLR3 as well as the fish-specific TLR22 (a cell surface TLR sensor) was observed in the spleen and kidney of TiLV-infected adult zebrafish and larvae (12–14). The upregulation of TLR3, normally present in cellular endocytic compartments, suggests its possible interaction with TIR-domain-containing adapter inducing interferon β (TRIF) to mediate the activation of NF-κB and the Interferon regulatory factor 3 (IRF3), leading mainly to the promotion of both inflammatory and IFN-β-mediated antiviral responses. Indeed, an abundant and upregulated expression of the gene transcripts encoding IRF3, a key transcriptional regulator of type I interferon (IFN)-dependent immune responses which plays a critical role in the innate immune response against DNA and RNA viruses, has been observed in the liver, spleen, intestine, gills and kidney of both tilapia and zebrafish infected with TiLV (12–14, 35).

In addition, a significant increase in IRF1 gene expression early during infection was observed in the liver of TiLV-infected tilapia (43). Similarly to IRF3, IRF1 is a member of the IRF family. Although it seems to not be essential for the induction of type I IFNs, IRF1 has nevertheless been found to play a significant role in IFN-mediated signaling, in TNF-mediated type I IFN signaling and in IFN-dependent inflammation (44). IRF1 protein can induce the expression of type I IFNs downstream of RLRs (45), and although IRF3 and IRF7 have always been assumed to be the predominant transcriptional regulators in the canonical TLR signaling, IRF1 has also nevertheless been shown to also participate in the transcriptional responses involving the engagement of some TLRs (such as TLR9, TLR7, TLR2, TLR3 and TLR4) or involving the myeloid differentiation primary response 88 (MYD88) protein. Increases in IRF1 expression induced by viral infections in most cases primarily result from NF-κB and STAT1-mediated transcriptional activation. IRF1 expression is usually induced rapidly following virus infection and there is evidence that IRF1 effector genes can suppress the replication of a variety of RNA viruses (46). Moreover, IRF1 can regulate basal antiviral states that restrict both positive- and negative-stranded RNA viruses in various cell types.

Likewise, IRF7 has also been found to be upregulated in both adult zebrafish and larvae (12–14). Both IRF7 and IRF3 promote the expression of genes encoding type I IFNs. IRF5 involved in the activation of the expression of type I IFNs and inflammatory cytokines downstream of endolysosomal toll-like receptors TLR7, TLR8 and TLR9 was also found to be upregulated (31), as well as the gene transcripts encoding for other interferon regulatory factors such as IRF4 and IRF8 (primarily involved in the adaptive immune response). Their possible role in adaptive immunity is discussed later in the section “*Activation and modulation of the T-cell adaptive immune response*”.

A significant upregulation of the PRR sensor RIG-I has also been reported in both adult zebrafish and larvae infected with TiLV (12–14). The signaling pathway triggered when RIG-I is activated is well characterised in mammals. After binding its nucleic acid ligands (RNA with a 5’ triphosphate moiety, uncapped short ssRNA or dsRNA), RIG-I signals via interaction of its caspase activation and recruitment domain (CARD) with an adapter protein associated with the outer mitochondrial membrane known as MAVS (mitochondrial antiviral signaling protein or IFN-β promoter stimulator (IPS)-1 protein) (47, 48). This CARD-dependent association of RIG-I and MAVS triggers a downstream transduction signaling cascade subsequently leading to the activation of IRF3 and IRF7 as well as NF-κB, thus causing the expression of a variety of type I IFNs and cytokines aimed at inhibiting viral replication (49). These mechanisms are also very likely to take place in teleost fish such as tilapia, especially when considering that RIG-I and MAVS (IPS) also exist in teleost fish (50–52), and their salmonid orthologues also have the same domain structures as seen in mammals (27).

In fact, MAVS (or IPS-1) has been found to be upregulated in the kidney and brain of tilapia fish during late stages (96 hours post-infection) of TiLV infection (42). In Atlantic salmon, a MAVS homologue protein (AsMAVS) has been found to mediate the activation of the salmon IFN-a1 promoter (53) although possessing the CARD, proline-rich and transmembrane domains found in mammalian MAVS. The observation that MAVS is also induced and upregulated during TiLV infection, indicates that MAVS may play a significant role in the RIG-I innate immune processes occurring during TiLV infection.

**Figure 1:**
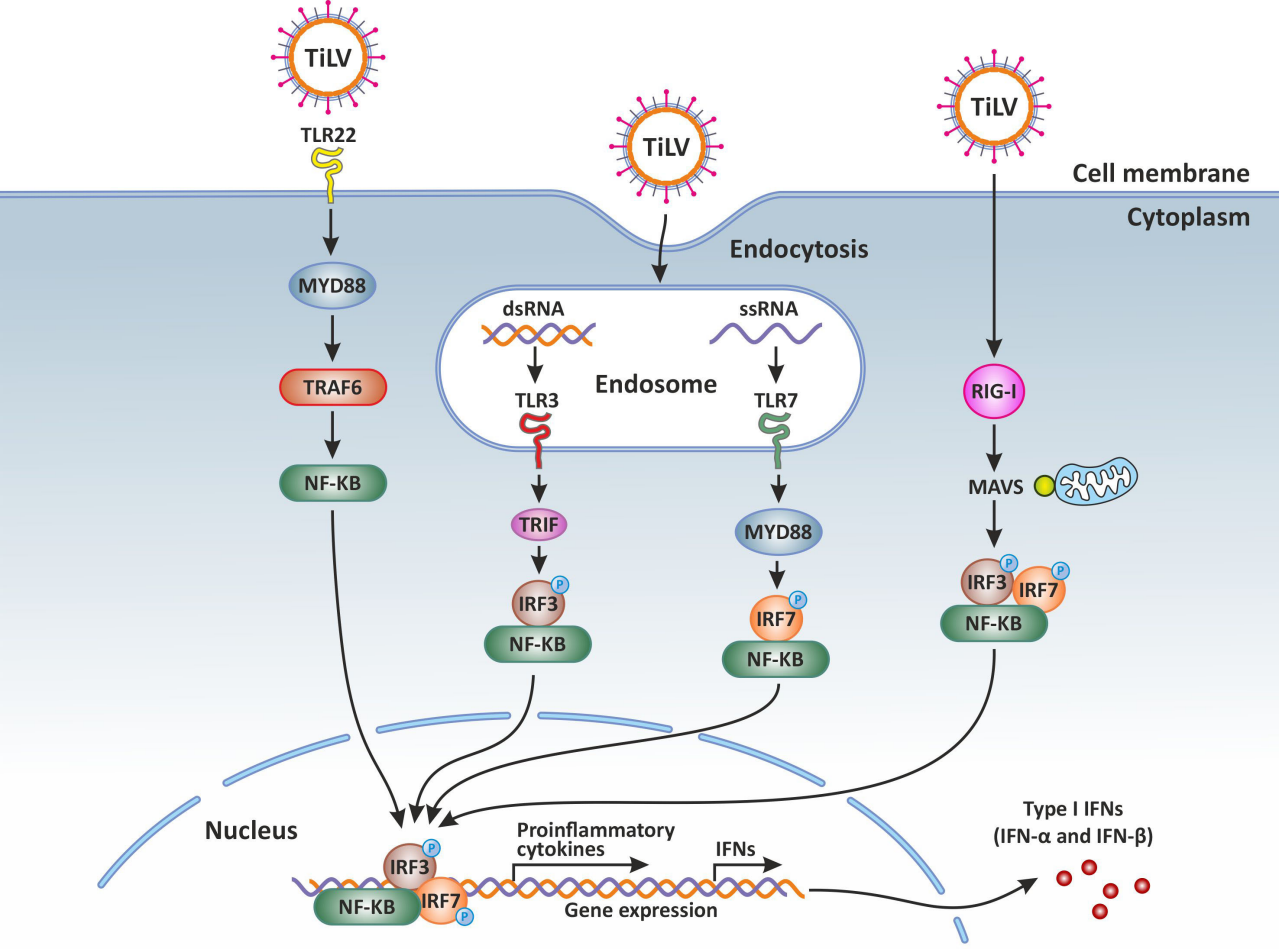
Innate immune response against Tilapia lake virus (TiLV) infection. Intracellular detection of TiLV infection by the host pathogen recognition receptors (PRRs) activates the transcription factors nuclear factor kappa-light-chain-enhancer of activated B-cells (NF-κB), interferon regulatory factor 3 (IRF3), and IRF7. The PRRs involved include toll-like receptors (3, 7 and 22) and retinoic acid-inducible gene-I protein (RIG-I). By signaling through the adaptor proteins MYD88 and TRIF, the activated transcription factors translocate to the nucleus and trigger the expression of type I and type III interferons (IFNs) which are crucial for the establishment of the antiviral state. Original image realized with CorelDRAW graphics suite 2020.

It has been shown that over-expression of MAVS in teleost fish protects cells from infection by both DNA and RNA viruses by inducing IFN stimulated genes (ISGs), such as IRF3 and the myxovirus resistance (Mx) as well as type I IFN (54, 55). The overexpression of MAVS protein could thus represent a potential therapeutic approach (56), for the treatment and prevention of TiLVD. Overall, PRRs sensors of the innate immune system and their adaptor proteins are activated during TiLV infection (**Figure 1**).

### 3.2 Antiviral molecules involved in innate immunity against TiLV infection

The activation of transcription factors such as NF-κB, IRF1, IRF3 and IRF7 during TiLV infection results in their translocation into the nucleus, where they initiate the transcription of genes encoding type I IFNs, inflammatory cytokines such as tumour necrosis factor (TNF)α, interleukins (IL-8, IL-1β and IFNγ1-2), and proinflammatory gene products such as cyclooxygenase-2 (COX-2) (31). Although the specific activation of NF-κB during TiLV infection has not yet been demonstrated, upregulation of both the tumour necrosis factor receptor associated factor 3 (TRAF3) and the nuclear factor-kappa-B-inhibitor alpha (NFKBIA) gene has been observed in the liver of TiLV-infected tilapia (31). TRAF3 is an adaptor protein that functions both independently as a negative regulator of the NF-κB pathway and as a positive regulator of type I IFN production. It is therefore at the intersection between the IFN-I and NF-κB pathways (57). The NFKBIA gene on the other hand, encodes for the alpha subunit of the IκB kinase (IKK) protein complex, which is a group of related proteins regulating the activity of NF-κB. The specific upregulation of these 2 factors regulating NF-κB function suggests the modulation of NF-κB downstream of the RIG-I sensing signaling pathway during TiLV infection. It will thus be interesting to determine how NF-κB is regulated during TiLV infection and if TiLV also induces an upregulation of the melanoma differentiation-associated protein (MDA5).

The significant upregulation of type I IFNs (*ifnϕ1*) during TiLV infection has been demonstrated in adult zebrafish and larvae (12–14). Moreover, treatment with exogenous recombinant zebrafish IFNϕ1 (zfIFNϕ1) has been shown to significantly decrease both the mortality and the viral load at 48 hours post-infection in TiLV-infected zebrafish larvae (14), suggesting the early administration of exogenous IFN as a therapeutic strategy. Indeed, it was recently shown that human IFN-α2a both completely prevented and inhibited TiLV infection (by more than 80%) when administered before the infection (58). Similarly, the fish IFNc significantly reduced TiLV-induced CPE and viral loads in a dose-dependent manner; further demonstrating the protective role of type I IFNs in preventing TiLV infection (58).

**Figure 2:**
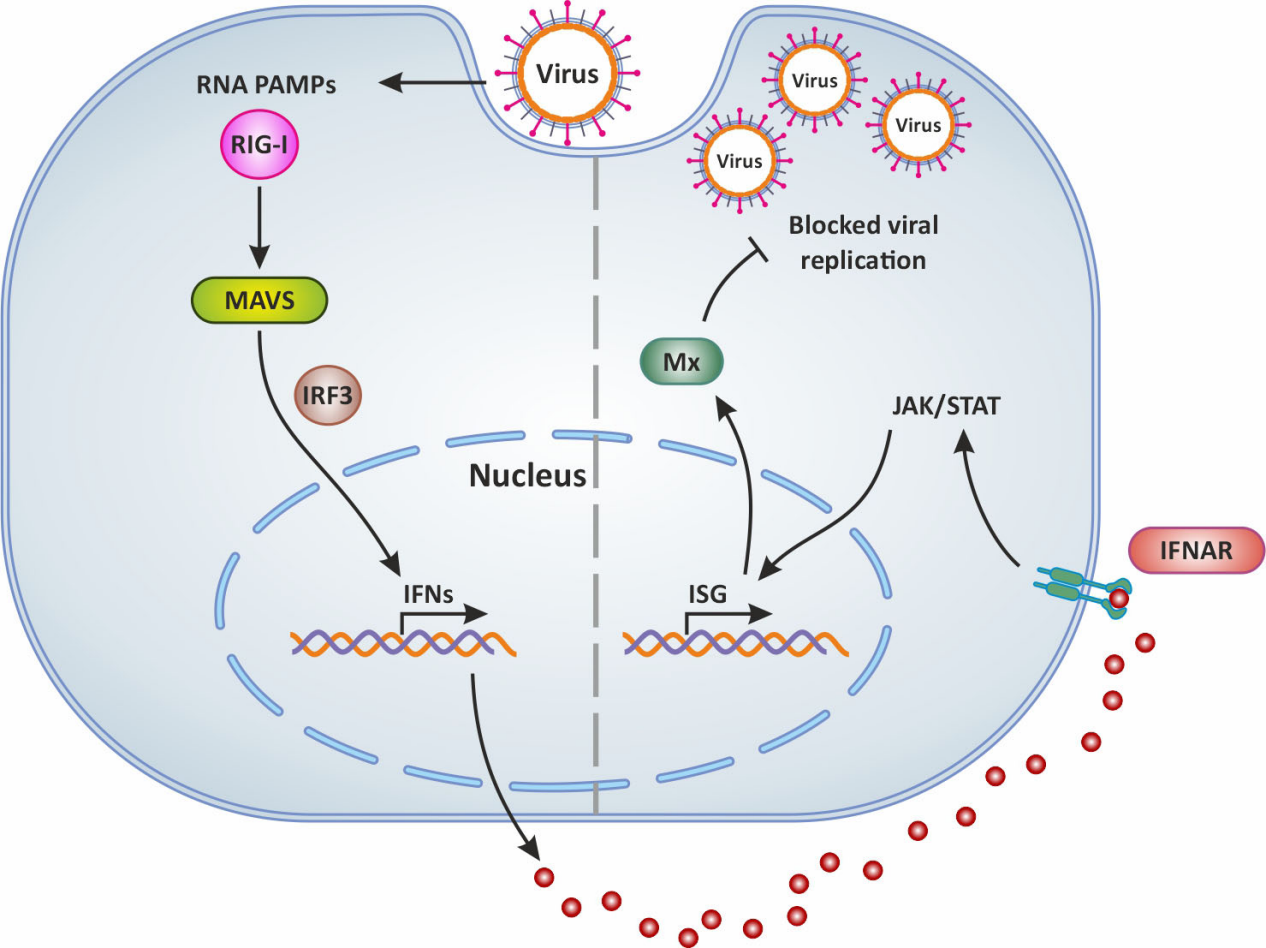
Mx production following virus infection and antiviral activity. In general, following virus infection, the RNA PAMPs of viruses activate RIG-I (which then activates the adaptor molecule MAVS downstream of RIG-I). This PRR signaling leads to the subsequent activation of the transcription factor IRF-3 for the induction of IFN. Secreted IFNs dock onto their receptor IFNAR and thus mediate the expression of antiviral ISGs via the JAK/STAT signaling pathway. This results in the production of Mx which blocks viral replication and is involved in IFN-mediated inhibition of viruses. Original image realized with CorelDRAW graphics suite 2020.

It is well known that type I IFNs act in autocrine and paracrine ways to induce the transcription of several ISGs, some of which encode antiviral proteins such as Mx (59, 60). The expression of genes encoding for Mx is controlled by type I interferons (61). Indeed abundant and significantly upregulated *mx* genes transcripts were detected in the brain, liver, spleen, intestine, and gills of TiLV-infected tilapia (31, 32, 35), and in adult and zebrafish larvae infected with TiLV (12–14) as well as brown and rainbow trout (15). Moreover, the administration of exogenous recombinant zfIFNϕ1 was found to up-regulate Mxa expression in zebrafish larvae infected with TiLV, which coincided with the observation of a significant reduction of TiLV viral load in zfIFNϕ1 pre-treated zebrafish larvae (14). Mx proteins are key components of the antiviral state induced by interferons. One unique property of some Mx GTPases is their antiviral activity (illustrated in **Figure 2**) against a wide range of RNA viruses, including orthomyxoviruses, paramyxoviruses, rhabdoviruses and members of the bunyavirus family. It has been shown for instance that the constitutive expression of Atlantic salmon (*Salmo salar*) Mx1 protein in CHSE-214 cells (fibroblastic cells deriving from Chinook Salmon - *Oncorhynchus tshawytscha* - embryo) conferred resistance to the cells against cytopathic infectious salmon anemia virus (ISAV) strain NBISA01. A resistance characterized by a delayed development of cytopathic effect (CPE), a significant reduction in the severity of CPE, and a 10-fold reduction in virus yield (62). An open question is thus whether or not TiLV is sensitive to the antiviral action of Mx.

Interestingly, the *rsad2* gene encoding for radical s-adenosyl methionine domain containing 2 protein, (also known as viperin), a multifunctional IFN-inducible protein, is also significantly highly upregulated in the liver of TiLV-infected tilapia (43). Although this IFN-inducible protein is constitutively highly expressed in the liver, its significant upregulation in liver cells of TiLV-infected tilapia suggests that viperin could be playing an important role in the regulation of TiLV infection cycle as this protein has been shown to inhibit a broad spectrum of DNA and RNA viruses, including herpesviruses, flaviviruses (Hepacivirus C [HCV], West Nile virus, and dengue virus), paramyxoviruses (Sendai virus and measles virus), a rhabdovirus (vesicular stomatitis virus), an alphavirus (Sindbis virus), a retrovirus (human immunodeficiency virus type 1, HIV-1) and an orthomyxovirus (Influenza A virus) (63). As an IFN-inducible protein, viperin is produced in a variety of cell types by stimulation with all types of IFNs and by infection with multiple viruses. Viperin induction by viruses is mediated by the classical ISG induction pathways involving the engagement of TLR3, TLR4 and RIG-1, which in turn activates IRF3 and IRF7. Alternatively, viperin can also be upregulated independently of IFNs by a number of viruses including human cytomegalovirus (HCMV), vesicular stomatitis virus, Japanese encephalitis virus, and Chikungunya virus (63).

**Figure 3:**
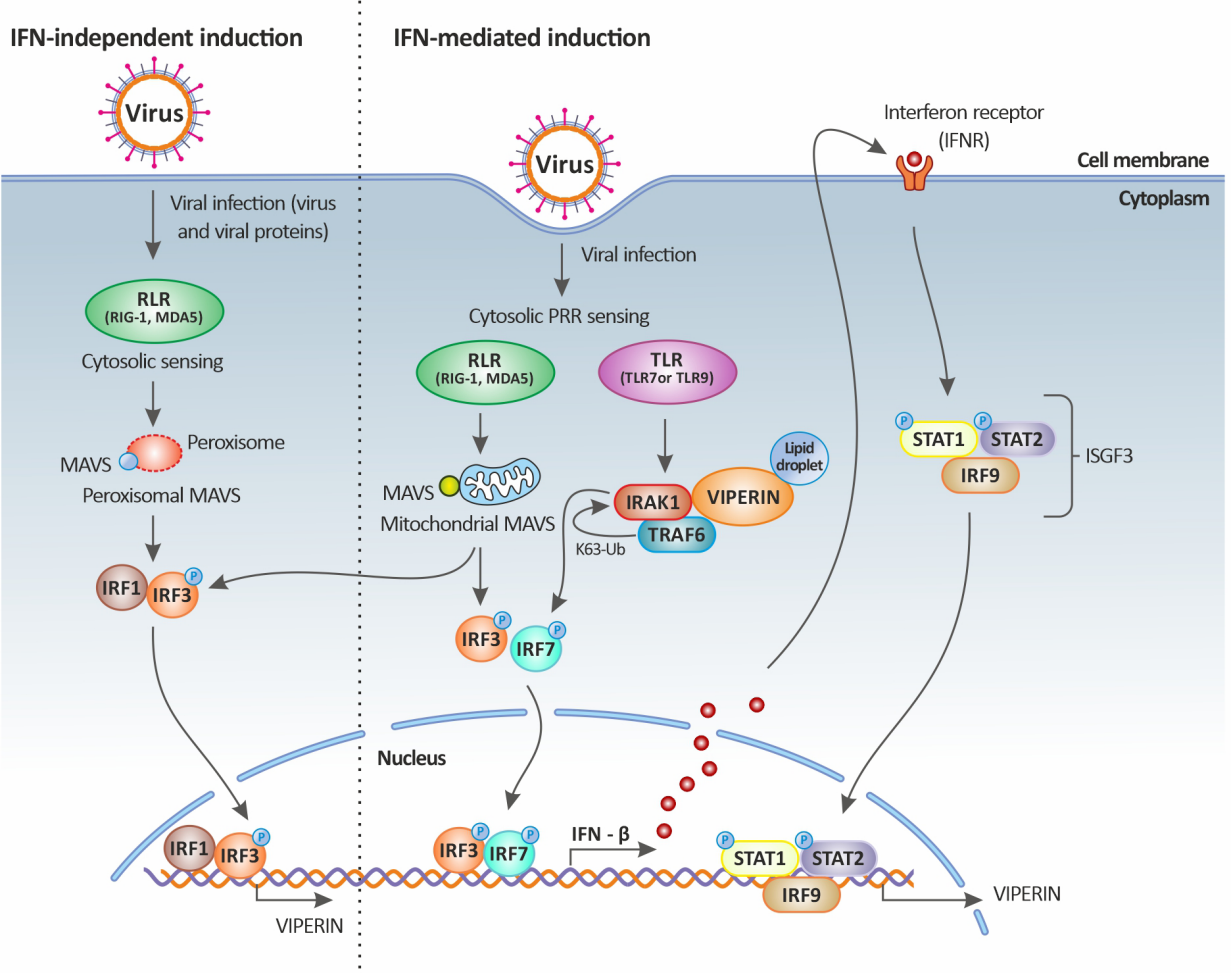
Viperin induction upon viral infection. Viperin expression is mediated by both the classical IFN-stimulated gene induction pathway (right panel) and the IFN-independent pathway (left panel). In the IFN-independent pathway, viperin gene expression is regulated by IRF1 and IRF3, which can be activated by viral factors or by the peroxisomal MAVS signaling pathway. In the IFN-mediated pathway, viperin gene expression is regulated by the ISGF3 complex. Upon viral infection, cytosolic sensing of viral nucleic acids by PRRs leads to the activation of downstream signaling factors including those dependent on endosomal TLRs and mitochondrial MAVS and culminates in the activation of the transcription factors IRF3 and IRF7. IRF3 and IRF7 translocate to the nucleus resulting in the induction of IFNs (more specifically IFN-β). The secreted IFN-β then signals both in autocrine and paracrine manners upon binding to its receptor, leading to the downstream activation of the Jak-STAT pathway. This in turn results in the formation of the heterotrimeric complex ISGF3, which translocates to the nucleus and binds to the promoter of ISGs, including that of viperin. Viperin itself can also increase IFN-β induction by promoting TRAF6-dependent ubiquitination of IRAK1 and phosphorylation of IRF7. Original image realized with CorelDRAW graphics suite 2020.

In fact, the direct stimulation of viperin expression can occur through the activation of MAVS following the downstream activation of IRF1 and IRF3. In this case, the stimulation of RLRs by dsRNA leads to the activation of the adaptor protein MAVS (or IPS-1) either residing on the peroxisome or at the mitochondrial membrane through an IRF1- and IRF3-dependent gene induction [illustrated in **Figure 3** and reviewed in (64)]. Thus, it appears that the significant upregulation of both IRF1 (43) and IRF3 (35) during TiLV infection, especially in the liver, could be leading to their translocation into the nucleus and their subsequent binding to the *rsad2* promoter (which contains functional binding sites for both IRF1 and IRF3). This in turn, leads to the induction of viperin in an IFN-independent manner; although the significant upregulation of type I IFNs [in adult zebrafish and larvae (12–14)] also raises the possibility that this protein might also be induced in an IFN-dependent manner.

The full antiviral mechanisms at play and associated with viperin antiviral functions against a wide range of viruses remain somewhat elusive, but overall range from inhibition of viral RNA replication, direct binding to viral proteins, to blocking of viral particles release (viral budding) by disruption of lipid rafts (65). However, some DNA viruses (notably the dsDNA herpesviruses HCMV and Kaposi’s sarcoma-associated herpesvirus - KSHV) appear to have repurposed the cellular roles of viperin to their benefit during viral replication. The first by co-opting viperin (HCMV) and the second by enhancing the activity of its viral protein (KSHV). Indeed, the co-option of viperin by HCMV alters the cellular metabolism which in turns favours HCMV replication. In the case of KSHV, the binding of viperin to the viral helicase protein enhances the stability of the protein and thus promotes viral replication (64). Therefore, can the remarkably high expression profile of viperin in the liver during TiLV infection (43), promote viral infection at this site or is it an attempt by host cells to restrict and inhibit viral replication? The severity of the infection in the liver [one of the main target organs of TiLV, hence the name syncytial hepatitis originally attributed to TiLVD (1, 23)] could make the first case scenario possible. Evidence is needed to draw any conclusions on the possible antiviral effects of viperin on TiLV replication.

It has been observed that TiLV infection induces an up-regulation of the expression of the gene encoding for pro-inflammatory cytokine IL-1β at mucosal sites such as the intestine and gills (35), as well as in the brain and liver of infected tilapia (31, 32, 66, 67). High expression levels of this mediator of inflammatory response have also been reported in both adult zebrafish and larvae experimentally infected with TiLV (12–14). IL-1β acts downstream of the nucleotide-binding domain leucine-rich repeat (NLR) family pyrin domain containing 3 (NLRP3) inflammasome (NLRP3) following binding to the IL-1 receptor (IL-1R). In fact, the activation of NLRP3 inflammasome contributes to the enzymatical maturation of the inactive precursor pro-IL-1β into its active form IL-1β (68). A signaling pathway thought to ensure the efficient secretion of IL-1β for the initiation of host innate immunity, which subsequently leads to the induction of both NF-κB-dependent inflammation and trafficking of neutrophils and T-cells (69). Similarly, an upregulation of the gene encoding for IL-8 was observed in the liver, spleen and head kidney of TiLV-infected tilapia (43, 70), and in both adult zebrafish and larvae infected with TiLV (12–14). IL-8 acts as a chemoattractant cytokine that specifically attracts and activates neutrophils in inflammatory regions (71). Therefore, the concerted action of IL-1β and IL-8 at infection sites may drive the massive infiltration of lymphocytic inflammatory cells that has been consistently observed in multiple organs including the brain, liver, intestines and spleen, during TiLVD (19, 35, 42, 43). The role of the NLRP3 inflammasome during TiLV infection and disease pathogenesis should thus be elucidated.

TNF-α is another inflammatory cytokine for which mRNA expression has been reported to be significantly upregulated during TiLV infection in both tilapia and zebrafish (12–14, 31, 66). TNF-α is produced by epithelial, endothelial and smooth muscle cells, as well as by astrocytes, activated macrophages, T and B lymphocytes, natural killer cells, and some tumour cells (72). It induces endothelial adhesion molecules, which trigger the migration of innate immune cells, such as blood-borne dendritic cells (DCs), natural killer (NK) cells and macrophages, to the site of infection. It is an interesting pro-inflammatory cytokine as it has been shown to inhibit the replication of viruses such as vesicular stomatitis virus, encephalomyocarditis virus, herpes simplex virus, influenza virus and HIV-1 in specific cell lines (72–74), but also stimulates HIV-1 replication in chronically infected T-cells and promonocytic cell lines (75–77). TNF-α also plays a crucial role in both the mitogen-activated protein kinases (MAPK) and the necroptosis (programmed necrosis) pathways. In the necroptosis pathway, the binding of TNF-α to membrane receptors TNFR1 activates the intracellular RIP (receptor interacting protein) family kinases. TNFR1 in turn interacts with the death domain of other adapter proteins, ultimately recruiting the receptor-interacting serine/threonine protein kinase 1 (RIPK1). RIPK1 and other proteins form a complex most often consisting of RIPK1, RIPK3, FADD and mixed lineage kinase domain like pseudokinase (MLKL), thereby activating MLKL. Activated MLKL then translocates to cellular membranes, causing their rupture (78).

This “dirty death” of cells i.e. necrosis, could be important in shaping disease evolution and pathogenicity as it enhances inflammatory reactions that may help curtail viral reproduction. Necrosis has been consistently associated with TiLVD pathology in several studies (1, 13, 19, 21, 24, 31, 32, 35, 37, 42). In addition, in TiLV-infected tilapia, several genes namely *tnfa*, *tnfrsf6b*, and *ripk1* all involved in the regulation of necroptosis have also been found to be upregulated (31). It is therefore possible that such a significant upregulation in *tnfa* and in genes involved in the regulation of necroptosis during TILVD progression both drive the development of programmed necrosis as observed in multiple organs, especially in the liver of TiLV-infected fish. It might thus be a host-induced strategy to both inhibit viral replication as previously reported (72–74) and enhance inflammatory reactions to lessen viral reproduction. It will therefore be crucial to elucidate the contribution of necroptosis in TiLVD pathogenesis. Furthermore, it will be interesting to determine if TNF-α inhibits or stimulates TiLV replication as previously described for other viruses and to determine in which specific cells this inhibition occurs, as this might be cell-type specific.

Chemokine genes, encoding for chemotactic key player cytokines controlling the migration of immune cells in tissues during the innate immune response to infections, have also been found to be upregulated in the liver of TiLV-infected tilapia, notably the chemokine (C-C motif) ligand (CCL3) together with its receptor CCR1 (31).

CCL3, produced by macrophages and implicated in macrophage, neutrophil and NK-cell migration as well as in T-cell – dendritic cells interactions, has been reported to be associated with antiviral immunity through the production of IFNγ, meaning that it is almost invariably associated with viral infections (79), although with few exceptions. Of note, the ifnγ1-2 gene was also found to be significantly upregulated in the brain of adult zebrafish during TiLV infection (12). Therefore, together with IFNγ, CCL3 could be driving the inflammatory response and phenotype in affected tissues, through the recruitment of CCR1 as previously described during paramyxovirus infection (80).

Genes coding for CXCR4, the specific receptor for the chemokine CXCL12, a highly potent chemoattractant involved in chronic inflammation (81) was also found to be upregulated in the liver of tilapia during TiLV infection (31). The production of CXCR4, is induced highly in the liver during HCV or Hepatitis B virus (HBV) infection and has been reported to be involved in directing immune cells from the circulation to the liver, while promoting their retention (81). It is therefore possible that CCL3 (possibly together with CXCL12) participates in driving the infiltration of lymphocytic inflammatory cells consistently observed in the liver during TiLV infection (21, 32, 35, 82). This is even more plausible when considering that genes encoding hematopoietic cell kinase - HCK [associated with enhanced secretion of pro-inflammatory cytokines (83)], and dedicator of cytokinesis - DOCK2 [reported to be critical for migration and activation of leukocytes (84)], both involved in inflammation and leukocytes migration, were also upregulated in the liver during TiLV infection (31).

### 3.3 Complement activation during TiLV infection

The complement system is a major component of the innate immune system. It consists of several plasma proteins responsible for various innate immune functions such as the elimination of invading pathogens, promotion of inflammatory responses, clearance of apoptotic cells and necrotic cell debris, and modulation of adaptive immune responses (85). Activation of complement leads to proteolytic cascades, terminating in opsonization and lysis of the pathogen as well as in the generation of the classical inflammatory response through the production of potent proinflammatory molecules. Almost all of the mammalian components of the complement system have homologues in teleost fish (27), and activation of complement generally occurs via three main pathways (classical, lectin, and alternative), depending on specific recognition molecules. It was recently found that genes coding for a significant number of components of the complement system such as C3, C4, C1R, CFB, CFD, C8A, C9, C1S, CFI and CFH, belonging to all three complement activation pathways, are upregulated in the liver of tilapia during TiLV infection (31). Moreover, genes encoding opsonins such as C-reactive protein (CRP) and signaling lymphocytic activation molecule (SLAM)-associated protein (SAP) which are involved in complement activation and facilitate the clearance of pathogens through phagocytosis (86) were also found to be upregulated during TiLV infection (31). Similarly, the gene encoding lysozyme LYZ, which is reported to stimulate the cellular and humoral defense mechanisms of fish and to provide protection against viral diseases (87), was also upregulated in addition to phospholipase A2 gene (*pla2s*) (31), which has been reported to block viral entry into cells (88). Furthermore, the gene encoding alpha2-macroglobulin (a2M), which has been reported to be involved in innate immunity against viruses and apoptosis (89), was also upregulated during TiLV infection (31).

### 3.4 Local innate immune response in the brain: TiLV induces brain inflammation and microglia activation

The consistent reports of TiLV infection and pathology in the brain (12, 16, 19, 32, 35, 42, 66, 82, 90), clearly demonstrate that TiLV exhibits neurotropism. When cells of the brain become infected, the rapid production of type I IFNs is important to ensure host survival, as it has been shown that mice lacking the receptor for these IFNs were more susceptible to fatal disease progression following Sindbis virus infection (91, 92).

As previously mentioned, genes encoding RIG-I, TLR3 and TLR7 are all upregulated during TiLV infection in the brain (12, 47), similarly to IRF3, IRF7 and MAVS (IPS-1) (12, 42). Interestingly, type I IFN genes (infϕ1) are also significantly upregulated in the brain of zebrafish IP-injected with TiLV (12). It is known that in mammals IFN-β is immediately and preferentially produced by neurons and glial cells of the brain during virus infection (93, 94), probably because of its reduced central nervous system (CNS) toxicity compared to IFN-α (93, 94). Moreover, IFN-β might induce the production of neurotrophic factors by astrocytes (95) and might also induce the local production of the anti-inflammatory cytokine IL-10 (96), which all participate in maintaining the integrity of brain cells by dampening the inflammatory response in the brain. In support of this, high levels of the gene encoding the anti-inflammatory cytokine IL-10 were also found in the brain of zebrafish during TiLV infection (12). However, probably in response to brain damage as a result of virus replication and as an attempt of the host to clear the infection, high levels of the genes encoding proinflammatory cytokines such as IL-1β, IFNγ1-2, TNF-α, IL-8 (*cxcl8a*), the enzyme COX2b, and the antiviral effector Mxa were also detected in the brain during TiLV infection (12, 66). This results in the induction of brain inflammation or encephalitis (12) reported during TiLV infection in the brain, probably driven by the concomitant potent inflammatory action of IL-1β and IL-8 cytokines on brain cells (71, 97).

Inflammatory cytokines such as TNF-α, IL-1β, and IFN-γ disrupt the blood-brain barrier (BBB) as well as the tight junction integrity of brain endothelial cells (98–100). These inflammatory cytokines signaling at the BBB during infection facilitate leukocytes trafficking into the CNS, which although essential for virus clearance (100, 101), has multiple consequences, including enhancement of inflammation and activation of microglia as also observed during TiLV infection (12).

Microglia are brain resident antigen presenting cells (APCs) and macrophages. They are involved in first line innate immunity of the CNS and have a large regulatory role in CNS immunity. They play an important role in controlling viral replication and reducing mortality in the early stage of infection (102). Activated microglia have a direct antiviral effect during viral infection by producing IFN-I after recognition of virus by PRRs, and the IFN produced by microglia exerts an indirect antiviral effect by acting on other cells. In addition, microglia can restrict viral infection by autophagy (103). In fact, microglia also affect the induction of the adaptive immune response in the brain, as it has been shown that the total number and percentage of activated CD4+ T-cells decrease, as well as the frequency and number of T regulatory cells significantly decrease following depletion of microglia (102), indicating that they are crucial for fully activating virus-specific T-cell responses.

Microglia activation during TiLV infection was characterized by a change in their shape from highly ramified cells in their resting state, to ameboid, spherical morphology when activated. Furthermore, genes expressing microglia markers such as *csf1r* and *cd68* in the brain of adult zebrafish and *apoeb* in the larvae were all upregulated further supporting their activation (12). However, phagocytosis or autophagy were not demonstrated. It thus remains to be determined whether such an activation also results in phagocytosis of TiLV-damaged brain cells. The ultimate questions however remain which specific cells of the brain (neurons, microglia, oligodendrocytes, meninges, astrocytes) are targeted by TiLV virus during its neurotropic stage of infection and what is the specific route of entry of TiLV virus into the CNS.

## 4 Adaptive immunity against TiLV infection

In general, the adaptive immune system recognizes foreign pathogens by means of two types of cellular receptors: the B-cell receptor (BCR) and the T-cell receptor (TCR). B- and T-cells are the main effector cells of the adaptive immune response. The adaptive immune response is regulated by several mechanisms and increases with antigen exposure, producing an immunological memory, which constitutes the basis of vaccine development. The adaptive response is generally established days after infection and can recognize specific foreign antigens, thus leading to a response that increases in both speed and magnitude with subsequent exposures (104). In general, B-cells mediate the antibody (humoral) responses while T-cells are mainly involved in cell-mediated immune responses. However, both the humoral and cell-mediated responses are essential in antiviral defense and the function of both arms occurs in concert.

The relationship between the innate and adaptive immune system occurs via the antigen-presenting cells (APC) such as dendritic cells (DCs) and macrophages which, after processing microorganisms, display the processed antigen molecules on their surface to be presented to T lymphocytes via the major histocompatibility complex (MHC) class 2 receptors, which in turn initiates the adaptive cell mediated immune response.

### 4.1 Induction of the adaptive immunity by activation of melano-macrophage centres

In teleost fish such as tilapia, antigen trapping and presentation occurs in melano-macrophage centres (MMCs), which often exist as complex discrete centres containing lymphocytes and macrophages (105). As such, they are thought to participate in the adaptive immune response, and they likely perform similar functions as mammalian germinal centers (GCs), although with certain differences (106). These immune-related structures, commonly seen within the reticuloendothelial supporting matrix of hematopoietic tissues, have been found to significantly increase in size and frequency in conditions of environmental stress and during infection (107, 108), and their proliferation is often associated with late stages of chronic infection (109, 110).

Throughout the progression of TiLVD, MMCs have been consistently found to increase in size and number in the liver and the spleen (36, 90), and in the kidney (82) of tilapia exposed to TiLV. Such an increase in MMC abundance is thus likely indicative of the activation of the adaptive immune system as the populations of lymphocytes (and macrophages) capable of mounting an immune response are often situated close to these sites of antigen trapping also associated with accumulations of melano-macrophages (27).

### 4.2 Cell-mediated adaptive immune response

Histocompatibility molecules are glycoprotein receptors encoded by a gene complex, which are expressed in almost all nucleated cells of the organism. MHC plays an important role for the presentation and recognition of both endogenous and exogenous antigens. In fact, antigen presentation is an important immunological process playing a crucial role in both the detection of viruses and virally infected cells by T-cells and the activation of cell-mediated adaptive immunity (111).

#### 4.2.1 Antigen presentation

As previously mentioned, antigens processed by cells are displayed on their surface via the MHC I and MHC II receptors to be presented to T-cells for cell-mediated immune response activation. MHC class I molecules are ubiquitously expressed while MHC class II molecules are expressed in specialized APCs such as DCs, B-cells and macrophages. While MHC class I molecules are expressed on the surface of all nucleated cells and present peptides to be recognized by the T cell receptor of CD8+ T-cells, MHC class II molecules are expressed by specialized immune cells and present peptides to CD4+ T-cells (111).

During TiLV infection, a couple of genes coding for proteins regulating and modulating MHC class I antigen presentation were found to be upregulated, especially in the liver of TiLV-infected tilapia fish (31). The *pmse2* gene, involved in altering the cleavage properties of the proteasome thereby enhancing MHC class I antigen presentation (112), was found to be upregulated during TiLV infection. Genes encoding heat shock proteins such as HSPA1s, HSPA4, HSPA5, and HSP90A, reported to serve as post-proteasomal peptide carriers delivering processed antigen peptides to transporters associated with antigen processing (TAPs) thereby preventing the degradation of processed epitopes by cytosolic peptidases (113), were also found to be upregulated during TiLV infection (31).

Similarly, genes encoding molecules involved in MHC class II pathway regulation such as the CD74 (I chain), which facilitates the assembly of alpha and beta subunits of the MHC II molecules within the endoplasmic reticulum (114), as well as GILT and CTSL, involved in reducing protein disulfide bonds formation thereby exposing epitopes for efficient MHC II-restricted binding and subsequent antigen presentation (115) and in the processing of class II-associated invariant chains followed by the loading of antigenic peptides into MHC II molecules (116) respectively, were all found to be upregulated during TiLV infection (31). The upregulation of these genes associated with and involved in MHC I and MHC II antigen presentation suggests that TiLV-deriving antigens and peptides are effectively processed, transported to the cell surface and presented to T-cells for the efficient induction of the cell-mediated adaptive immunity.

### 4.3 Activation and modulation of the T-cell adaptive immune response

Mature T-cells possess a T-cell receptor (TCR) by which they recognize linear antigens presented by MHC molecules. They express the TCR co-receptor CD8 or CD4 which drives their specificity for MHC class I or MHC class II presented antigens respectively, while also having the potential to form immunological memory in case of future pathogen insult.

There is a clear distinction between CD8 and CD4 expressing T cells, based on the expression of the co-receptors CD8 or CD4 (CD8+ and CD4+ cells respectively). While CD8 marks cytotoxic T lymphocytes (CTLs) that recognize antigenic peptides associated with MHC class I molecules on the surface of antigen presenting cells and whose main function is the direct killing of target cells. CD4 on the other hand, marks T helper cells (Th cells) that recognise peptides associated with MHC class II and which orchestrate several aspects of the adaptive immune response via the release of modulatory cytokines.

The specific activation and cytotoxic actions of CD8+ cells during TiLV infection have not yet been demonstrated. However, at later stages during TiLV infection (at 6 to 14 days post-infection), a significant up-regulation of the expression of CD4 markers *cd4-1* and *cd4-2* was observed in the liver and spleen of TiLV-infected zebrafish (13), suggesting the activation of CD4+ and their cell-mediated antiviral action. The role of CD4+ T-cells in antiviral immunity is highly dependent on the production of pro-inflammatory cytokines such as IFN‐γ (79, 117), which has also been found to be significantly upregulated in the spleen and kidney of zebrafish during TiLV infection (13). CTLs are major producers of IFN‐γ, which is also the hallmark cytokine of Th1 cells activation. By also producing IFN‐γ, Th1 cells, which are crucial for controlling most viral infections, promote CTL-mediated lysis of infected target cells by stimulating the maturation of CTL precursors. In addition, IFN‐γ stimulates the production of several other proteins that contribute to enhanced antigen presentation and T-cell activation including MHC molecules.

It is also possible that the significant up-regulation of genes encoding the anti-inflammatory cytokines tumour growth factors (TGF)-β and IL-10 in the intestine, spleen, liver and kidney of TiLV infected tilapia and zebrafish (13, 35, 67) aims at promoting the development of Th17 cells, although IL-6 and IL-1 might also be required; the presence and up-regulation of which have not yet been demonstrated during TiLV infection. Such an upregulation in TGF-β and IL-10 levels might also predict the activation of T-reg cells since these two cytokines are also produced by T-reg cells. Although Th17 differentiation is inhibited by IFN‐γ and IL-4, DCs can also efficiently present antigens to Th17 in the presence of TGF-β, thereby promoting Th17 cell differentiation. However, TGF-β and IL-10 remain natural anti-inflammatory cytokines which, together with T-reg cells, can strongly suppress immune responses to pathogens.

Other gene transcripts encoding for other interferon regulatory factors involved in adaptive immunity have also been found to be upregulated in the liver of tilapia fish infected with TiLV. This is for instance the case of IRF4 (31), thought to regulate the maturation and differentiation of immune cells, especially the development of effective cytotoxic T-cell responses during viral infection (118) and IRF8 (31), which is involved in CD8+ dendritic cell differentiation and is required for natural killer (NK)-cell-mediated antiviral immunity by promoting the proliferation of virus-specific NK cells (119). As previously mentioned, the gene transcripts coding for *viperin* (*rsad2*) are also significantly upregulated in the liver during TiLV infection (43). Such an upregulation in the expression of viperin suggests that this IFN-induced protein may also be involved in regulating Th2 cells response (120) during the infection and could thus be modulating anti-TiLV T-cell-mediated immunity.

#### 4.3.1 Adaptive immunity activation in the brain

During most neurotropic viral infections in mammals, the activation of naive T-cells and B-cells occurs in secondary lymphoid tissues outside of the CNS (121–123), although there exist cells that can present antigens to primed T-cells in the CNS. Although the route of entry of TiLV into the CNS remains to be determined, inoculation of neurotropic viruses directly into the cerebrospinal fluid (CSF) tends to elicit a potent immune response characterized by marked antibody responses and priming of cytotoxic CD8^+^ T-cells (124). Both tight junctions and the relative nonreactivity of cerebral capillary endothelial cells generally restrict the entry of circulating leukocytes into the CNS. However, it has been shown that activated T-cells can routinely cross the blood–brain barrier as part of the normal immunological surveillance of all tissues (125–127) and can be retained in the CNS when the relevant antigen is present and associated with appropriate MHC molecules (125).

Indeed, infiltration of mononuclear inflammatory immune cells into the CNS can occur days following neurotropic virus infections, with cells first accumulating in the perivascular areas, as also observed during TiLV infection in the brain (16, 90), followed by massive infiltration in the regions of virus infection, which has also been described during TiLV infection (32, 42, 82). Such a massive infiltration can drive the occurrence of lymphocytic meningitis, a condition often associated with brain inflammation, and which has also been described during TiLV infection (19).

It will thus be of interest to elucidate if the local production of chemokines during brain inflammation caused by TiLV infection induces the expression of adhesion molecules by endothelial cells, which could enhance the entry of activated immune cells into the CNS. Of particular interest, the expression profiles of the genes encoding the intercellular adhesion molecule 1 (ICAM1, CD54) and the vascular-cell adhesion molecule 1 (VCAM1) during TiLV infection could be investigated as this could provide insights into the immune cell infiltration observed during TiLV infection, and open avenues for lymphocytic entry blockade using antibodies or ligands binding to these molecules.

Microglia activation has also been reported during brain inflammation following TiLV infection (12), whether such an activation, in turn, leads to brain DCs differentiation remains to be elucidated.

### 4.4 Humoral (antibody) response during TiLV infection

As previously mentioned, B-cells mediate antibody (humoral) responses. During antibody responses, B-cells are activated to secrete antibodies, which are soluble forms of their surface immunoglobulin (Ig) antigen receptor. The antibodies circulate in the bloodstream, binding specifically to the foreign antigen that stimulates their production. The binding of antibodies to their targets such as viruses, results in their inactivation and the blocking of their ability to bind to their receptors on host cells. In addition, antibody binding also tags invading pathogens for destruction by cells such as phagocytic cells of the innate immune system bearing cell surface receptors for the Ig molecules.

**Figure 4:**
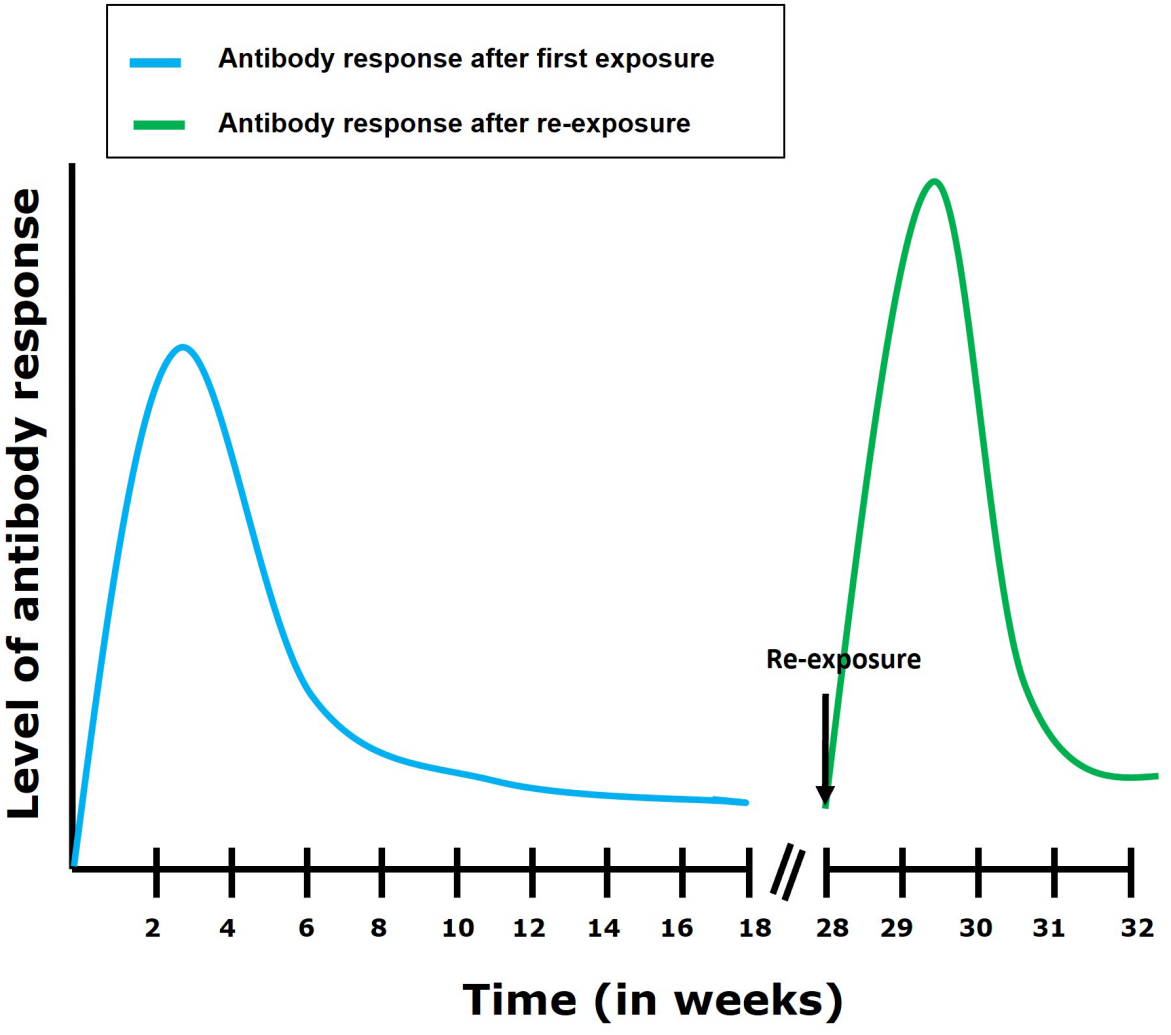
Antibody response following first exposure and re-exposure to TiLV infection. It has been shown (29) that the antibody response in most tilapia fish following first exposure to TiLV reaches its highest level within 4 weeks post exposure. The antibody levels then gradually declines but remains maintained till about 16 weeks post-exposure. After re-exposure, an elevated and more rapid (7 to 14 days to establish itself) antibody response can be observed which then gradually declines.

During TiLV infection, it has been shown that tilapia can mount a humoral antibody response reaching high levels within 2 to 4 weeks post primary exposure to the virus (29). This antibody response is characterized by an upregulation of IgM mRNA levels in organs such as the brain, head kidney, liver (in tilapia), spleen and kidney (in zebrafish) of TiLV infected fish (13, 32). In some individuals, circulating antibodies persisted for up to 110 days in TiLV-exposed tilapia; upon re-exposure, an antibody response was shown to develop within 7 to 14 days (**Figure 4**) (29). Interestingly, some individuals only produce antibodies against TiLV about 12 weeks post-primary exposure to the virus. Moreover, few tilapia have been found to survive both the initial exposure and a subsequent TiLV virus challenge without generating an antibody response (29). This suggests a great variation in disease susceptibility and probably immune defense mechanisms which could be characterized by the induction of a robust innate immune response which could have resolved the infection without inducing the adaptive immunity in these individuals. Alternatively, it is also possible that these individuals are naturally resistant to TiLV infection. Factors associated with disease resistance are thus also discussed later.

Strikingly, fish specific IgT antibody production, which is associated with mucosal immunity in teleost fish (128, 129), has not yet been reported following TiLV natural infection. Moreover, the gene transcripts encoding this antibody isotype (*igt 1/2*) were downregulated in the liver, gill and brain of TiLV-resistant tilapia fish strains and only slightly upregulated in the brain of a TiLV-susceptible strain later during infection (67). Interestingly, a significant upregulation of this antibody isotype was observed in the head kidney of tilapia fish vaccinated with heat-inactivated whole TiLV virus (130), suggesting that IgT might still be produced at mucosal surfaces during infection with live virus. Moreover, accelerated host responses in mucosa could be related to significantly lower viral loads in a TiLV-resistant tilapia strain (67), probably indicating that mucosal immunity also plays an important role in protection from TiLV infection.

The role of mucosal immunity during TiLV infection should warrant further studies, which will also enable the mechanisms governing specific B-cells activation during TiLV infection to be uncovered. The full spectrum of antibodies produced during infection with TiLV should also be explored as this could reveal a great deal about humoral immunological responses against viral infections in teleost fish such as tilapia in general.

## 5 Interplay between TiLV and host responses

### 5.1 Host factors associated with resistance to TiLV infection

Several significant quantitative trait loci (QTL) affecting resistance to TiLV were identified on chromosomes Oni3 and Oni22 of farmed Nile tilapia (*Oreochromis niloticus*). The average mortality rate of tilapia fish homozygous for the resistance allele at the most significant single nucleotide polymorphism (SNP) on these QTLs was 11% compared to 43% for tilapia homozygous for the susceptibility allele. Several candidate genes related to the host response to viral infection were identified within these QTLs, including *cdc42*, *trim21* and *trim29* (131).

In mice, CDC42 appears to be a key regulator of B-cell differentiation required for antiviral humoral immunity (antibody responses), germinal center formation, and formation of plasma cells during influenza virus (PR8 strain) infection (132). Moreover, *cdc42* gene was also mapped to a QTL associated with host resistance to cardiomyopathy syndrome (133) in Atlantic salmon populations. The role of CDC42 as a key regulator of B-cell fate and physiology during TiLV infection should thus be explored.

The ubiquitin ligase tripartite motif containing-21 (TRIM21) antibody receptor provides one of the last lines of defense against invading viruses by acting as a sensor that intercepts antibody-coated viruses (virus neutralization) that have evaded extracellular neutralization and breached the cell membrane (134). TRIM29 on the other hand has been found to negatively control antiviral immune response to DNA viruses (135). Moreover, TRIM29 has been found to inhibit innate immune activation following Epstein-Barr virus infection (136) and to negatively regulate type I IFN production during dsRNA reovirus infection by interacting with MAVS and by inducing its K11-linked ubiquitination and degradation (137).

Interestingly, the gene coding for TRIM21 was found to be significantly upregulated in the liver, gill and brain of TiLV-susceptible tilapia strains but only in the liver and gill of a TiLV-resistant tilapia strain during TiLV infection (67). However, resistance to TiLV disease in this resistant tilapia strain was correlated with lower viral loads both at the mucosa-rich tissue of the gills and internal tissues, once more highlighting the need for studies to understand the role of mucosal immunity during TiLV infection. Moreover, a higher magnitude of Mx-1-based antiviral response possibly limiting virus spread in the initial phase of infection was proposed as a possible mechanism driving such lower viral load as well as the lower pro-inflammatory responses exhibited by the TiLV-resistant strain, which is thought to additionally contribute to its protection from developing pathological changes related to the disease (67).

The resistance of red hybrid tilapia to TiLV infection could also be modulated using probiotic-supplemented diets, which resulted in lower cumulative mortality rate and significantly lower viral load (in the liver, spleen and head kidney), especially in red hybrid tilapia fed with 1% *Bacillus spp* probiotics. In addition, in tilapia fed with 1% *Bacillu*s *spp*, a significant upregulation of the expression of *inf-γ* and *il-8* genes was observed in the liver, spleen, and head kidney subsequently contributing to improving the antiviral response mounted by this group of fish against TiLV (70).

### 5.2 Possible immune subversion by TiLV virus

It has been shown that TiLV can downplay the innate immune responses during the early stage of infection in Nile tilapia (32). Although the exact mechanisms by which TiLV subverts and modulates the immune response remain to be elucidated, it has been observed that, following TiLV infection, there is a downregulation of genes encoding both the interferon-induced proteins with tetratricopeptide repeats (IFIT1) (in interferons) and TRIM25 (in the NF-kB pathway) in the liver of TiLV-infected tilapia (31), further suggesting that TiLV is capable of modulating the host immune response to its advantage to establish and sustain the infection.

Indeed, IFIT1 is strongly induced downstream of type I IFN signaling. Although the mechanisms underlying the antiviral activity of IFITM proteins in general remain uncertain, IFIT1 could exert one of its antiviral activities by recognizing and potentially sequestering viral triphosphorylated RNA (PPP-RNA), thereby preventing it from being translated by the host machinery (138). In addition, it has been shown that human IFIT1 can suppress IRES-dependent viral RNA translation during HCV infection (139). It has also been shown to directly bind to viral proteins such as the human papillomavirus (HPV) viral helicase E1 protein required for HPV viral replication, leading to E1 sequestration within the cytoplasm, thus preventing it from aiding in viral replication within the nucleus (140, 141).

On the other hand, the E3 ubiquitin ligase TRIM25 has been shown to play a role in the RIG-I pathway, triggering the expression of type I interferons upon viral infection. TRIM25 has been shown to inhibit influenza A virus (IAV) infection by destabilizing IAV viral mRNA, and its direct tethering to an RNA molecule is also sufficient to downregulate the targeted RNA (142). TRIM25 could also inhibit flavivirus and birnavirus replication *in vitro* (143, 144) and in the case of birnavirus infection, by targeting VP3 for ubiquitination and degradation (144).

The downregulation of such antiviral restriction factors suggests possible immune evasion and modulation strategies of TiLV to successfully establish and sustain the infection within its host. Immune evasion and modulation strategies by the virus should therefore warrant further investigations.

## 6 Discussion

### 6.1 Knowledge gaps

It is clear that many questions remain to be addressed regarding the immune responses of tilapia to TiLV infection. The exact virus entry site(s) remain to be determined as well as the specific immune responses at those entry sites. The mechanisms by which TiLV escapes the early immune responses at entry sites, and which lead to its systemic dissemination [and systemic infection - (43)] also remain unresolved.

TiLV has been shown to persist in the brain (for up to 90 days) during infection (12). Such a long viral persistence in the brain suggests that the brain is an immune privileged site incapable of clearing the infection. Ideally, the development of antibodies (especially neutralising antibodies) during the infection, should provide sterile immunity against the pathogen. However, the persistent detection of the virus in asymptomatic tilapia could indicate that the virus can hide from the immune system even at late stages of the infection. The immune evasion mechanisms governing this persistence of the virus as well as the lack of virus clearance in the brain also remain largely unknown.

Infection with TiLV is in most cases lethal. However there have been reports of fish surviving the infection (67, 90, 131) suggesting the existence of specific immune mechanisms leading to both favourable (preventing mortality) and unfavourable (immunotoxic) disease outcomes, both of which are still to be established.

TiLVD outbreaks are often detected within complex diseases involving the virus and additional pathogens, most often bacteria and parasites. Moreover, TiLV has been associated with co-infections with other viruses such as tilapia parvovirus (TiPV) (145). Although the synergism between TiLV and TiPV coinfection remains to be determined, this could suggest that TiLV is passively (by disrupting mucosal barriers) or actively (by inducing immunosuppression) promoting superinfections, thus worsening disease severity. However, the mechanisms of mucosa disruption or immune subversion by TiLV infection remain to be clarified.

### 6.2 Closing remarks

Since the initial reports of TiLV disease and infection in 2014 (23, 90), several studies have been undertaken to elucidate the immune responses occurring during TiLV infection. Although these studies, and the immune response branches they address (all summarized in **Table 1**), have greatly contributed to our knowledge of the possible immune responses of tilapia to TiLV infection, it remains clear that the specific mechanisms underlying the antiviral response to TiLV infection are still poorly studied and understood. For instance, the nature of the specific cells involved in innate immunity against TiLV infection remains to be determined as well as the exact mechanisms by which TiLV suppresses and subverts the host immune response to establish the infection. The full spectrum of antibodies generated during TiLV infection remains to be established, and the exact mechanisms of TiLV entry into the CNS remain to be uncovered as well as the specific brain cells targeted by the virus.

**Table 1:** Summary of the different studies addressing the pathology and immune responses to TiLV infection.

Immune component	Immune response	References
**Pathogenesis**	Syncytia formation	(1, 11, 17, 19, 23, 24, 35–37, 43)
	Necrosis	(1, 13, 19, 21, 24, 31, 32, 35, 37, 42)
	Upregulation of genes involved in the regulation of necroptosis, in inflammation and in leukocyte migration	(31)
	Infiltration of lymphocytic inflammatory cells in the brain, liver, intestines and spleen	(19, 21, 32, 35, 42, 43, 82)
**Innate immunity**	Upregulation of *tlr3*, *tlr7*, *tlr22* in tilapia and zebrafish	(12–14, 42)
	Upregulated expression of *irf1*, *irf3*, *irf5*, *irf7* in tilapia and zebrafish	(12–14, 31, 35, 43)
	Upregulated expression of *rig-1* in zebrafish	(12–14)
	Upregulated expression of *mavs* (*ips-1*) in tilapia	(42)
	Upregulation of *traf3* and *nfkbia* genes in tilapia	(31)
	Upregulation of type I IFNs (*ifnϕ1*) in zebrafish and antiviral activity of type I IFN against TiLV infection	(12–14)
	Upregulation of *mxa* gene transcripts in tilapia and zebrafish	(12–14, 31, 32, 35)
	Upregulation of genes coding for *mx1* and *rsad2* (viperin) transcripts in brown trout and rainbow trout	(15)
	Upregulation/high levels of IL-1β in tilapia and zebrafish	(12–14, 31, 32, 35, 66, 67)
	Upregulation of *il-8* in tilapia and zebrafish	(12, 43, 70)
	Upregulation of *tnf-α* in tilapia and zebrafish	(12, 13, 31, 66)
	Upregulation of chemokine *ccl3* gene and chemokine receptor *cxcr4* gene/Upregulation of genes coding for components of the complement activation pathways/Upregulation of opsonins genes in tilapia	(31)
	Upregulation of *rsad2* (viperin) expression in tilapia	(43)
	TiLV infection and pathology in the brain	(12, 16, 19, 32, 35, 42, 66, 82, 90)
	Induction of brain inflammation (encephalitis)/Microglia activation	(12)
**Adaptive immunity**	Activation of MMCs in tilapia	(36, 82, 90)
	Upregulation of genes encoding proteins regulating or modulating MHC class I antigen presentation/Upregulation of genes involved in MHC class II pathway	(31)
	Upregulation of the expression of CD4 markers (*cd 4-1* and *cd 4-2*)/Upregulation of IFN‐γ	(13)
	Upregulation of *irf4* and *irf8* in tilapia	(31)
	Accumulation of mononuclear inflammatory immune cells at perivascular areas in the brain	(16, 90)
	Massive infiltration of immune cells in the brain/Lymphocytic meningitis	(19, 32, 42, 82)
	Upregulation of *igm* in tilapia and zebrafish	(13, 32)
	Downregulation of IgT (*igt 1/2*) in resistant tilapia strains and upregulation in susceptible tilapia strain	(67)
	Upregulation of *tgf-β* and *il-10* in tilapia and zebrafish	(13, 35, 67)
	Development of protective immunity including a humoral response after the exposure to TiLV	(29)
**Interplay between TiLV and host responses**	Downregulation of *ifit1* and *trim25*	(31)
	Host factors located in the vicinity of QTL associated with resistance to TiLV infection (*cdc 42*, *trim21, trim29*)	(131)
	Host factors associated with responses during resistance to TiLV infection (upregulation of *mx1*, downregulation of *il1b*)	(67)

The presence of TiLV in the intestine (13, 34) raises the notion of TiLV antigen sampling in the intestine, which we believe should also be addressed, since it could significantly improve our knowledge of the development of the adaptive cellular and humoral response in the intestine (which could be one of the main port of entry for the virus).

We have seen that resistance to TiLV disease in some tilapia strains correlates with lower viral loads at the mucosa-rich tissue of the gills (67) and that accelerated host responses in mucosa could be related to such significantly lower viral loads in a TiLV-resistant tilapia strain (67). It thus becomes clear that the extent of the role played by mucosal immunity during TiLV infection control should be further addressed.

The complex network regulating both the innate and adaptive immune responses during TiLV infection remains to be uncovered and explored. It will be interesting to know whether similar mammalian Th1, Th2, Th17 and Treg responses occur during TiLV infection and if these responses are governed by mechanisms similar to those in their mammalian counterparts. Moreover, the T-cell and B-cell interplay should be explored to understand their implications in immunopathogenesis.

An overall downregulation of genes involved in cellular metabolism has been observed during TiLV infection (31). The implications of such a downregulation on the overall immune profile of the host tilapia during the infection should also thus be explored.

## Author contributions

JK-R: Conceptualization, methodology, investigation, resources, writing and review – original draft. DS: review and editing – original draft. KT: review and editing – original draft. JD: supervision, review and editing – original draft. MA: supervision, funding acquisition, resources and review and editing – original draft. All authors contributed to the article and approved the submitted version.
